# Rifampin-associated tubulointersititial nephritis and Fanconi syndrome presenting as hypokalemic paralysis

**DOI:** 10.1186/1471-2369-14-13

**Published:** 2013-01-16

**Authors:** Hong Ki Min, Eun Oh Kim, Sang Ju Lee, Yoon Kyung Chang, Kwang Sun Suh, Chul Woo Yang, Suk Young Kim, Hyeon Seok Hwang

**Affiliations:** 1 Division of Nephrology, Department of Internal Medicine, The Catholic University of Korea, Seoul, Korea; 2 Department of Pathology, Chungnam National University College of Medicine, Daejeon, Korea

**Keywords:** Rifampin, Fanconi syndrome, Tubulointerstitial nephritis, Hypokalemic paralysis

## Abstract

**Background:**

Rifampin is one of the most important drugs in first-line therapies for tuberculosis. The renal toxicity of rifampin has been reported sporadically and acute tubulointerstitial nephritis (ATIN) is a frequent histological finding. We describe for the first time a case of ATIN and Fanconi syndrome presenting as hypokalemic paralysis, associated with the use of rifampin.

**Case presentation:**

A 42-year-old man was admitted with sudden-onset lower extremity paralysis and mild renal insufficiency. He had been treated for pulmonary tuberculosis with isoniazid, rifampin, and ethambutol for 2 months. Laboratory tests revealed proteinuria, profound hypokalemia, hyperchloremic metabolic acidosis with a normal anion gap, positive urine anion gap, hypophosphatemia with hyperphosphaturia, hypouricemia with hyperuricosuria, glycosuria with normal serum glucose level, generalized aminoaciduria, and β2-microglobulinuria. A kidney biopsy revealed findings typical of ATIN and focal granular deposits of immunoglubulin A and complement 3 in the glomeruli and tubules. Electron microscopy showed epithelial foot process effacement and electron-dense deposits in the subendothelial and mesangial spaces. Cessation of rifampin resolved the patient’s clinical presentation of Fanconi syndrome, and improved his renal function and proteinuria.

**Conclusion:**

This case demonstrates that rifampin therapy can be associated with Fanconi syndrome presenting as hypokalemic paralysis, which is a manifestation of ATIN. Kidney function and the markers of proximal tubular injury should be carefully monitored in patients receiving rifampin.

## Background

Rifampin is one of the most effective antibiotics used for the treatment of tuberculosis. The renal toxicity of rifampin has been reported sporadically and acute renal failure is a frequent clinical presentation [1]. Histologically, rifampin nephrotoxicity is associated with acute tubulointerstitial nephritis (ATIN), tubular necrosis, papillary necrosis, acute cortical necrosis, and minimal change disease [1-3]. Of these, ATIN and tubular necrosis are the most common and frequently develop upon reintroduction of the drug or during intermittent therapy.

Acquired Fanconi syndrome occurs at any age as an autoimmune disorder or after exposure to noxious agents [4,5]. It is characterized by a generalized transport defect in the proximal tubules and can be associated with drug-induced ATIN [6-8]. However, neither Fanconi syndrome nor associated hypokalemic paralysis has ever been reported in patients with rifampin-associated ATIN. We describe a case of ATIN and Fanconi syndrome presenting as hypokalemic paralysis in a patient receiving rifampin.

## Case presentation

A 42-year-old man was hospitalized with sudden-onset weakness in both lower extremities. The patient had been treated with isoniazid 300 mg, rifampin 450 mg, and ethambutol 800 mg daily for pulmonary tuberculosis during the past two months. He had no history of exposure to antituberculosis antibiotics before beginning therapy. His compliance to antibiotics was excellent after the treatment for pulmonary tuberculosis and he denied the interruption on these regimens. He reported no constitutional symptoms, history of hyperthyroidism or paralysis.

A physical examination indicated that the muscle strength of the lower extremities was 2/5 of normal. The rest of the physical examination was unremarkable. A chest radiograph revealed reticular opacities on the bilateral upper lung fields, consistent with pulmonary tuberculosis. His laboratory data included hemoglobin 13.0 mg/dL, leukocyte count 9900/μL, and platelet count 290,000/μL. His biochemical data for serum and urine on admission are shown in Table 1. Profound hypokalemia, hypophosphatemia, and hypouricemia were present. Serum pH, bicarbonate level and anion gap indicated hyperchloremic metabolic acidosis with normal anion gap. The urinary anion gap was positive (10.2 mEq/L), suggesting the presence of renal tubular acidosis. Urinalysis showed normoglycemic glucosuria, β_2_-microglobulinuria, pH 5.5, 2.5 g/day proteinuria and microscopic hematuria. The fractional excretion of potassium was 29.3% (normal range, 4%–16%); the calculated ratio of the maximal tubular transport of phosphate reabsorption to the glomerular filtration rate (TmP/GFR) was 0.24 mg/dL (normal range, 2.3–4.3 mg/dL); and the fractional excretion of uric acid was 77.4% (normal range, 6%–20%). Generalized hyperaminoaciduria was detected with liquid chromatography–tandem mass spectrometry. These findings suggest generalized proximal tubular dysfunction with wasting of bicarbonate, glucose, protein, potassium, phosphate, and uric acid.

**Table 1:** Biochemical Data on Admission and after Rifampin Withdrawal

	**Admission**	**2 weeks**	**3 months**	**6 months**	**8 months**
**Serum**					
Urea nitrogen (mg/dL)	13.9	-	18.5	15.9	15.9
Creatinine (mg/dL)	1.4	-	1.12	1.18	1.09
Potassium (mmol/L)	2.0	3.8	3.8	3.7	4.3
pH	7.289	7.260	7.387	7.381	7.367
Bicarbonate (mmol/L)	12.4	14.5	22.8	23.3	24.4
Phosphate (mg/dL)	1.2	2.7	5.2	4.4	4.6
Uric acid (mg/dL)	1.2	1.3	3.9	4.5	4.2
Sodium (mmol/L)	141	140	140	140	142
Chloride (mmol/L)	114	112	103	103	106
Magnesium (mg/dL)	2.1	2.3	2.5	2.3	2.3
Albumin (g/dL)	4.7	-	-	4.3	4.3
**Urine**					
24-h glucose (g/day)	16.90	-	0.05	0.06	-
β_2_-microglobulin (μg/L)^*^	>20000	-	1238	265	-
Potassium (mEq/L)	7.2		29.5	61.4	67
Phosphate (mg/dL)	11.7		33.7	69.5	96.8
Uric acid (mg/dL)	11.4		28.1	-	67.3
Creatinine (mg/dL)	17.2		38.99	189.7	171
FE_K _(%)^†^	29.33	-	22.29	10.32	9.80
TmP/GFR (mg/dL)^‡^	0.24	-	4.23	3.96	3.98
FE_UA _(%)^§^	77.41	-	20.69	-	10.2
pH	5.5	6.0	5.0	5.0	5.5
24-h protein (g/day)	2.50	0.91	0.10	0.14	0.01

Note: Replacement of potassium chloride and sodium bicarbonate was interrupted at 3 months after admission.

^*^Normal, less than 370 mg/L.

^† ^Normal, 4-16%.

^‡^Normal, 2.3 to 4.3.

^§^Normal, 6-20%.

FE_K_, fractional excretion of potassium; TmP/GFR, tubular maximal transport of phosphate reabsorption to the glomerular filtration rate transport; FE_UA_, fractional excretion of uric acid.

Additional blood tests were performed to determine other possible causes of the patient’s hypokalemia. His plasma renin activity and serum level of aldosterone were 21.6 ng/mL/h and 30.5 ng/dL, respectively. His thyroid-stimulating hormone and free thyroxine levels were within the normal ranges. Serum and urinary protein immunoelectrophoresis showed no evidence of monoclonal gammopathy, and immunological surveys of autoantibodies were negative. The patient’s reticulocyte count, serum lactate dehydrogenase, and liver enzymes levels were within the normal ranges.

**Figure 1:**
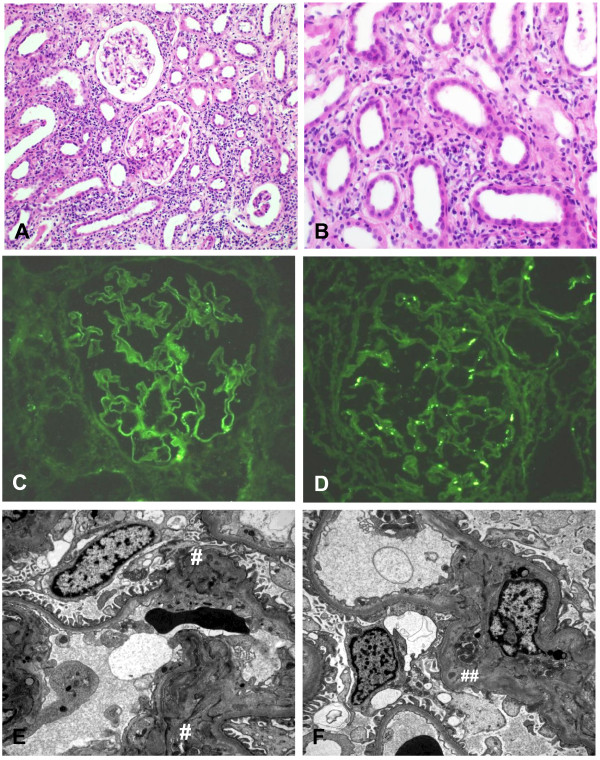
**Pathologic findings in a patient with rifampin**-**associated acute tubulointerstitial nephritis and Fanconi syndrome. **Light microscopy revealed the extensive mononuclear cell infiltrates including epithelioid histiocytes and eosinophils, mild interstitial fibrosis and tubular atrophy (**A**: original magnification X 100; **B**: original magnification x 400). Immunofluorescent stains showed focal granular deposits of immunoglobulin A (**C**) and complement 3 (**D**) in mesangial spaces and tubules. Subendothelial electron-dense deposits (**E**) and expanded mesangial spaces with electron dense deposits (**F**) were visible using electron microscopy.

An ultrasonographic examination showed a normal-sized kidney, with slightly increased echogenicity in the bilateral renal parenchyma. A renal biopsy showed extensive mononuclear cell infiltrates, including epithelioid histiocytes and eosinophils, mild interstitial fibrosis, and tubular atrophy (Figure 1A and B). Ziehl–Neelsen staining for acid-fast bacilli and PCR detection of *Mycobacterium tuberculosis* in the renal biopsy specimen were negative. Focal granular deposits of immunoglobulin A (IgA) and complement 3 (C3) were demonstrated in the tubules (Figure 1C and D). The same immunofluorescent positivity was also shown in glomerular mesangium, and electron microscopy showed electron-dense deposits in the subendothelial and mesangial spaces (Figure 1E and F). These findings were consistent with IgA nephropathy accompanied by focal immune deposits along the tubules. Half the epithelial foot processes were effaced, and there were no pathological findings in the mitochondria.

Potassium chloride supplementation was given to treat the patient’s hypokalemic paralysis. His muscle strength increased one day after potassium chloride supplementation. We substituted rifampin with levofloxacin, but his other antituberculosis antibiotics remained the same. Supplementation with potassium chloride and sodium bicarbonate was continued, and the patient was discharged on hospital day 14. The biochemical markers associated with Fanconi syndrome were significantly resolved, and he experienced no paralytic symptoms after discharge. His proteinuria and microscopic hematuria was also improved, and we discontinued supplementation with potassium chloride and sodium bicarbonate at the three-month follow-up. The patient’s renal function and proximal tubular function remained stable at the last follow-up.

## Discussion

We have reported for the first time rifampin-associated ATIN and Fanconi syndrome presenting as hypokalemic paralysis. The typical findings of ATIN were present in a kidney biopsy specimen from the patient, and generalized proximal tubular dysfunction was demonstrated by his biochemical parameters. The symptoms of Fanconi syndrome disappeared after the discontinuation of rifampin, which strongly indicates that rifampin was the causative agent of Fanconi syndrome.

The clinical manifestations of rifampin-associated nephrotoxicity can be mainly classified into two groups, depending on the anti-rifampin antibody induced [9,10]. In patients treated with an interrupted regimen, the rifampin-dependent antibody produces acute tubular necrosis requiring dialysis, intravascular hemolysis, and flu-like symptoms. In contrast, the continuous administration of rifampin has been described as progressing more insidiously. In this setting, tests for anti-rifampin antibody are uniformly negative, and the degree of interstitial inflammation is much greater than that of acute tubular necrosis [11]. Our patient had no history of prior exposure to rifampin or interruption on regimen, and reported no constitutional symptoms. Furthermore, the results of a kidney biopsy were compatible with the latter condition. These findings suggest that ATIN occurred independently of anti-rifampin antibodies, and the absence of intravascular hemolysis further supports this view.

Several types of drugs cause ATIN-induced Fanconi syndrome, and mitochondrial dysfunction, direct tubular toxicity, and immune reactions have been considered to be the pathogenic mechanisms [4,12,13]. In this patient, a renal biopsy showed no structural abnormalities of the mitochondria, but demonstrated extensive lymphocytic infiltration with eosinophils. Immunofluorescent staining also showed focal deposits of IgA and C3 along the tubules. These findings suggest that hypersensitive immune-mediated injury to the tubular membrane was the pathogenic mechanism of Fanconi syndrome, and anti-tubular basement membrane antibodies have been suggested to induce ATIN in such cases [10-14].

The coexistence of glomerular and tubular lesions is relatively rare in drug-induced ATIN. In our patient, the same immunofluorescent positivity shown in the damaged tubules was demonstrated in the glomerular mesangium, and electron-dense deposits were also present in the subendothelial and mesangial spaces. These findings suggest a relationship between the tubular insult and the immune injury progressing to the glomeruli. We infer that the release of tubular antigens as a consequence of rifampin-induced immunological injury gave rise to superimposed immune complex deposition in the glomeruli, which progressed further to IgA nephropathy [15-17].

Hypokalemia is usually mild to moderate in Fanconi syndrome [18]. However, profound hypokalemia with muscle paralysis was the presenting feature in this patient. The mechanisms of renal potassium wasting were considered to be as follows. First, the reduced reabsorption of potassium in the proximal tubule increased the urinary potassium loss. Second, the bicarbonaturia resulting from the impaired bicarbonate reabsorption in the proximal tubule enhanced the renal potassium excretion [19]. Third, the increased aldosterone level in our patient suggested that the reduced proximal sodium reabsorption increased the distal delivery of sodium, resulting in the depletion of the extracellular fluid volume and secondary hyperaldosteronism [20].

The management of drug-induced ATIN includes the immediate discontinuation of the offending agent and the use of corticosteroids [12,13]. A short course of corticosteroid is usually administered to patients with rifampin-induced ATIN, and severe renal failure, prolonged renal failure, or crescentic nephritis increases the need for corticosteroids [21,22]. In our case, cessation of the offending agent without corticosteroid restored the patient’s renal function and resolved his proteinuria. Furthermore, the clinical and biochemical presentations of Fanconi syndrome were completely reversed. Therefore, we suggest that the discontinuation of rifampin alone can improve the ATIN and Fanconi syndrome in such patients, when the patient shows insidious clinical progress without serious renal failure or crescentic nephritis.

## Conclusion

In conclusion, this case illustrates that rifampin can be associated with ATIN and Fanconi syndrome, presenting as hypokalemic paralysis. Therefore, Fanconi syndrome should be considered in the spectrum of renal diseases associated with rifampin. Renal function, in particular proximal tubular function, should be closely monitored when rifampin is used.

## Consent

Written informed consent was obtained from the patient for publication of this case report.

## Abbreviations

ATIN: Acute tubulointerstitial nephritis; C3: Complement 3; FE_K_: Fractional excretion of potassium; FE_UA_: Fractional excretion of uric acid; IgA: Immunoglobulin A; TmP/GFR: Tubular maximal transport of phosphate reabsorption to the glomerular filtration rate transport.

## Competing interests

The authors declare that they have no competing interests.

## Authors’ contributions

HKM, EOK, SJL, YKC, SYK, and HSH treated the patient and provided data about the history and laboratory results in this report. HSH performed the renal biopsy. KSS interpreted the kidney biopsy. The manuscript was prepared by HKM, CWY, and HSH. All authors participated in discussions about the manuscript and approved the final version.

## Pre-publication history

The pre-publication history for this paper can be accessed here:

http://www.biomedcentral.com/1471-2369/14/13/prepub
